# A Review of the Commercially Available ECG Detection and Transmission Systems—The Fuzzy Logic Approach in the Prevention of Sudden Cardiac Arrest

**DOI:** 10.3390/mi12121489

**Published:** 2021-11-30

**Authors:** Michał Lewandowski

**Affiliations:** 2nd Department of Arrhythmia, National Institute of Cardiology, 04-628 Warsaw, Poland; mlewandowski@ikard.pl; Tel.: +48-601174549

**Keywords:** sudden cardiac arrest, sudden cardiac death, out-of-hospital cardiac arrest, ischemic brain damage, prevention, cardiopulmonary resuscitation, electrocardiogram (ECG), fuzzy logic, artificial intelligence, detection algorithms

## Abstract

Sudden cardiac death (SCD) constitutes a major clinical and public health problem, whose death burden is comparable to the current worldwide pandemic. This comprehensive review encompasses the following topics: available rescue systems, wearable electrocardiograms (ECG), detection and transmission technology, and a newly developed fuzzy logic algorithm (FA) for heart rhythm classification which is state-of-the art in the field of SCD prevention. Project “PROTECTOR”, the Polish Rapid Transtelephonic ECG to Obtain Resuscitation for development of a rapid rescue system for patients at risk of sudden cardiac arrest (SCA), is presented. If a lethal arrhythmia is detected on the basis of FA, the system produces an alarm signal audible for bystanders and transmits the alarm message along with location to the emergency medical center. Phone guided resuscitation can be started immediately because an automated external defibrillator (AED) localization map is available. An automatic, very fast diagnosis is a unique feature of the PROTECTOR prototype. The rapid detection of SCA is based on a processor characterized by 100% sensitivity and 97.8% specificity (as measured in the pilot studies). An integrated circuit which implements FA has already been designed and a diagnosis is made within few seconds, which is extremely important in ischemic brain damage prophylaxis. This circuit could be implemented in smart implants (Sis).

## 1. Introduction

Sudden cardiac death (SCD) is a major clinical and public health problem. It remains one of the greatest challenges in cardiology and medicine, being one of the most terrifying situations in everyday life and clinical practice. Sudden cardiac arrest (SCA) and SCD refer to the sudden cessation of cardiac activity with hemodynamic collapse. The term SCA relates to a situation when an intervention (e.g., defibrillation) or spontaneous reversion restores circulation. If the patient dies, the event is called SCD. However, the use of the term “SCD” to describe both fatal and nonfatal cardiac arrest persists by convention. The detailed definitions for sudden death and aborted cardiac arrest are presented in 2015 European Society of Cardiology (ESC) Guidelines for the management of patients with ventricular arrhythmias and the prevention of SCD and other documents [1,2,3]. The SCD is an unexpected sudden death which occurs within 1 h of symptom onset due to a cardiac cause. The specific causes of SCA vary with the population studied and patient age. The outcome following SCA depends on numerous factors, including the underlying cause and the promptness of cardiopulmonary resuscitation (CPR) commencement. Most individuals suffering from SCA become unconscious within seconds to minutes as a result of insufficient cerebral blood flow. There are usually no prodromal symptoms and if they are present, they are nonspecific and include chest discomfort, palpitations, shortness of breath, and weakness. Events are witnessed by at least one person in only two out of three cases, making the diagnosis difficult to establish in many instances. It is estimated that, on average, less than 10% of all patients with SCA will survive. Time is key in CPR. Five top messages from contemporary guidelines concerning CPR include: (1) recognize cardiac arrest, (2) start CPR, (3) alert emergency medical services, (4) start chest compressions, (5) get an automated external defibrillator (AED) [4].

The abovementioned 2015 ESC Guidelines define gaps in this field. Simple and cheap methods appropriate for mass screening for sudden death risk are needed. More research is necessary to evaluate optimal design of “chain of survival”, including pre-hospital care. It is recommended that a public access defibrillation system should be established (Class I, Level B) [1].

The author believes that the review presented below and the research project based on telemedicine fulfills these requirements and its implementation may revolutionize the results of CPR.

## 2. Ischemic Brain Damage and Clinical Importance of the SCA

Cardiac arrest results in an instant cessation of perfusion through all organs and tissues. It leads to rapid oxygen deficiency and acidosis in all cells of the body and the dying process begins. It affects different tissues at different rates depending on their oxygen demand. This means that some vital organs may be already irreversibly damaged by ischemia while others may still tolerate impaired oxygenation. The brain is the tissue most vulnerable to ischemia. Within only three minutes after cessation of blood, its cortex and subsequently other important structures are damaged. Neuronal ischemic dysfunction can be fully reversed only at very early stages of cardiac arrest with prompt CPR. Any delays in CPR are related to poorer neurological prognosis and increased incidence of coma. In patients with SCA, rapid CPR is essential to shorten the time of central nervous system hypoxia. Only this kind of fast response provides better treatment outcomes, reducing the chance for disability in such patients in the future. Resuscitation attempt is generally reported in 67–82% of cases and success rate, i.e., survival to a hospital discharge, is achieved in approximately 2–40% of cases [5,6,7,8,9,10].

Clinical presentation of such patients after SCA may vary between impairment of cognitive function throughout persistent vegetative state (PVS) to severe brain stem injury. The long-term prognosis of people suffering from severe post-resuscitation anoxic-ischemic encephalopathy is usually bleak. Most of these patients require life support (such as mechanical ventilation, hemodynamic support, renal replacement therapy) and treatment in intensive care units (ICU). Others need to stay in long-term care facilities. The one-year mortality rate in both groups is very high. Effective chain of survival (early recognition, early resuscitation, early defibrillation, post-resuscitation care) remains the most important determinant of neurological outcome after SCD [1,2,3,4].

## 3. Epidemiology and Pathogenesis

Death certificate data suggest that SCD accounts for approximately 15–20% of the total mortality in the USA and other industrialized countries [6]. In absolute terms, the estimated out-of-hospital sudden cardiac arrest (OHCA) number in the USA was 356,000 in 2019 and a similar number in Europe. There are estimates for four million SCDs worldwide every year. The OHCA is the third most common cause of death in the USA [11,12,13,14,15].

**Figure 1:**
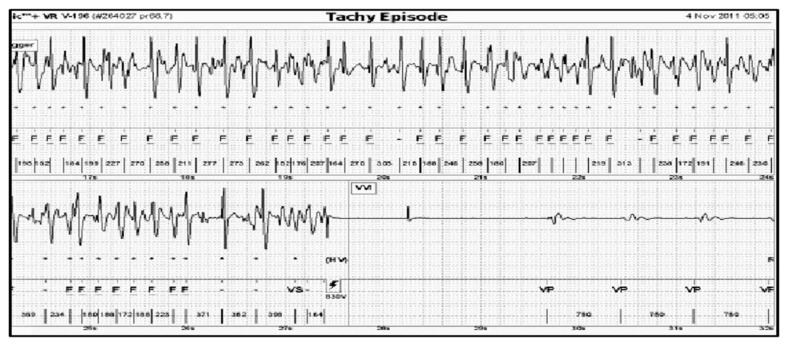
ICD-stored electrogram during an episode of ventricular fibrillation terminated with high voltage shock (HV).

The predominant mechanism of SCA is ventricular arrhythmia (84%) in the form of sustained pulseless ventricular tachycardia (VT) or ventricular fibrillation (VF) (Figure 1). The pulseless electrical activity (PEA) or bradyarrhythmia (mostly asystole) lead to SCA in the rest of the cases. These events occur mostly in patients with structural heart disease (that may not have been previously diagnosed), particularly coronary heart disease, cardiomyopathies, etc. and their consequences, or in a structurally normal heart with an underlying inherited arrhythmia disease [14,16,17].

## 4. SCA Management

Survival of patients with OHCA has been essentially unchanged, averaging 7.6% for all OHCA and 17.7% for OHCA due to VF in recent years [18]. During the last decade, the time from cardiac arrest to the start of CPR and defibrillation has increased, whereas survival has remained unchanged due to slightly better results of CPR [12,19]. Resuscitation of OHCA patients is based on the “chain-of-survival” concept, including early access, CPR, defibrillation, advanced cardiac life support, and post-resuscitation care. Bystander-initiated CPR is known to improve survival outcomes [4,10]. Thus, several organizations like the International Liaison Committee on Resuscitation (ILCOR), the European Parliament, and the World Health Organization (WHO) [11,12] recommend that CPR training should be included in secondary school curriculum in order to increase the number of people with Basic Life Support (BLS) skills, which in turn improves the rate of bystander-performed CPR. Such classes are already mandatory in several primary and secondary schools and universities across Europe, USA, and other countries [20].

### 4.1. How to Recognize Cardiac Arrest?

The correct early diagnosis is a crucial issue in such a situation. Slow, labored breathing (agonal breathing) should be considered a sign of cardiac arrest. A short period of seizure-like movements can occur at the beginning of this event. The affected person should be assessed after the seizure has stopped: if they are unresponsive and with absent or abnormal breathing, CPR should be performed. Failing to recognize cardiac arrest remains a barrier to saving more lives.

### 4.2. Harm from CPR to Victims Not in Cardiac Arrest?

Lay people may be reluctant to perform CPR on an unresponsive person with absent or abnormal breathing because of concern that delivering chest compressions to a person who is not in cardiac arrest could cause serious harm. The evidence for harm from CPR to victims not in cardiac arrest was reviewed by ILCOR in 2020 [11]. The scarcity of published reports on such harm strengthens the arguments that harm is likely very rare and desirable effects will far outweigh undesirable ones. ILCOR recommends that laypersons initiate CPR for presumed cardiac arrest without concerns of harm to patients not in cardiac arrest. Chest compressions (100–120/min, optimal ratio to breaths 30:2) as a part of CPR are essential in the management of SCA. A recent review and meta-analysis identified nine papers evaluating video instructions for simulated OHCA [14]. Compression rates were better with such instructions, and there was a trend towards better hand-placement [4,21].

### 4.3. How to Alert the Emergency Services?

Emergency medical services (EMS) must be alerted immediately if a person is unconscious and with absent or abnormal breathing. A lone bystander with a mobile phone should dial the EMS number, activate the speaker or another hands-free option on the mobile phone, and immediately start CPR assisted by the dispatcher. If a lone rescuer has to leave a victim to alert the EMS, they should call EMS first and commence CPR immediately after.

### 4.4. AED

Although an analysis of data from large registries shows that only a few percent of OHCA patients were treated by bystanders with an AED, recent data have shown possible benefit of public access defibrillators [22,23,24,25]. The main factors related to a better prognosis of patients with OHCA are shockable rhythm, witnessed cardiac arrest, resuscitation provided by a witness, and rapid response of EMS. Attempts to improve survival of patients with OHCA should be focused on identification of high-risk patients prior to an occurrence of SCA, increased implementation of bystander CPR training, better identification of high-incidence sites and more frequent usage of AED (via mobile phone), analysis of factors which can shorten the time of EMS response, and improved therapy of CSA victims [26,27,28].

### 4.5. AED Locator Apps

In the case of OHCA, early defibrillation increases the chance of survival; therefore, retrieving an AED during an emergency is crucial. Nevertheless, it can be challenging because the rescuer needs to know where the nearest AED is located. Thanks to built-in global positioning systems (GPS) in smartphones, numerous apps have been developed to locate the user and display the location of the nearest AEDs. The registry of AEDs in the vicinity and the route to reach the arrhythmic event location is crucial for integrated emergency help systems. During phone-guided CPR, dispatchers can locate and alert first-responder persons who are in the immediate vicinity of an OHCA patient through a text message or activate a smartphone app and guide the responder to the nearest AED and offer consultation for the first rescuer in real time [28,29].

### 4.6. Recent SCD Achievements

Recently, evident progress in protecting the population against SCD has been observed [14]. The advancements made in screening, prevention, and treatment, including underlying causes, have decreased the numbers of deaths. From 1980, implantable cardioverter–defibrillator (ICD) devices are in use and from 2010, the subcutaneous implantable cardioverter–defibrillator (S-ICD), the powerful preventive technology, has been implemented. To this date, these devices are the mainstay of SCD prevention, and the growing number of implantations has been a major factor in SCD decline of the number of SCD events [30]. Nevertheless, we must remember the complications of this therapy, especially in so-called primary prophylaxis. Related complications include inappropriate shocks, device and lead damages, pocket infections, endocarditis, psychological sequelae, or even therapy-related death [1]. An alternative for an implantable device is an external vest defibrillation (Zoll) but it is recommended only as a temporary solution bridging for cardiac transplant or ICD implantation [31]. The cost of this system is extremely high (around USD 3370/month to lease). The effect of drugs in SCD prevention is disappointing except for beta blockers and, to a lesser degree, amiodaron. Some antiarrhythmic drugs (quinidine, mexiletine, and flecainide) are beneficial in some quite rare disorders like Brugada syndrome, Long QT syndrome (LQTS), and catecholaminergic polymorphic VT (CPVT). The genome-wide association (GWAS) studies have provided new biological insights into the genetic link to SCD. This genetic contribution identified some genes: *KCNQ1*, *KNCH2*, *SCN5A*, *CACNA1C*, *CACNAB2B*, mutations in ryanodine receptors *RyR2*, and mutations in *MYBPC* or *MYH7* and locus *BAZ2B,* which are correlated with SCD risk [14]. An autopsy in the case of sudden death is essential. It has a crucial role in deceased family member risk stratification [32]. There is a progress in OHCA treatment but the overall survival rate of SCA is low and estimated to be still around only 10%. Therefore, the SCD prevention and not the treatment is the main goal. After a successful CPR, the treatment strategies include cardiac revascularization, catheter arrhythmia ablation, targeted temperature management (TTM, also known as induced hypothermia), and venoatrial extracorporeal membrane oxygenation (VA-ECMO). The treatment details are beyond the scope of this review. There are some future directions worth mentioning. One of them is extracorporeal CPR (eCPR) outside the hospital. In this approach, a portable extracorporeal membrane oxygenation (ECMO) machine is used as an adjunctive therapy to standard CPR [33,34].

## 5. How Technology Can Help in SCA Management

Modern technology is used in everyday activities, from our smartphones, computers, intelligent homes, and cars, etc., to innovative applications in medicine. Several projects have been developed encompassing different areas of technology implementation in CPR and BLS. There is growing interest among researchers in integrating smartphones and smartwatches in education and training regarding CPR and defibrillation, and for improving the response to OHCA [35]. The main trends are smartphone applications as a basic aid for bystanders providing help for OHCA patients. The CPR feedback in real-time communication between a medical professional and a lay bystander seems to be extremely useful. The new “sci-fi” technology allows the dreams of the potential use of drones and artificial intelligence (AI) in the chain of survival come true. Several studies have investigated the feasibility of delivering AEDs with drones to a simulated OHCA scene without problems during drone activation, take-off, landing, or bystander retrieval of the AED from the drone, and confirmed that AEDs could be expected to arrive earlier by drone than by ambulance [36,37].

## 6. Commercially Available ECG Tracking Systems

There are many commercially available electrocardiogram (ECG) measurement and transmission systems with mobile applications on paired mobile devices (smartphone, tablet, etc.) [37]. Their implementation in clinical practice is still far from being perfect nor cheap. Three types of these systems with ECG transmission are widely used: (1) smartwatches (e.g., I-Watch by Apple, [38]), (2) different kinds of I-Wear with this feature on the chest or extremities and the systems with the electrodes attached to the skin (e.g., Viatom Monitor ECG, DUOEK wearable EKG monitor with the chest straps etc., which cost from about USD 50 up to USD 300) [39,40], (3) systems requiring pressing two fingers onto a dedicated sensor (e.g., KardiaMobile by Alivcor; price: EUR 155€) [41]. All mentioned solutions called—“medgagets”—require checking the connection after initiating each use. One of the highly regarded products on the market is Spanish Nuubo, a patient textile ECG monitoring device with a monthly cost of USD 295 [42]. This system consists of a textronic lead embedded in the vest and an ECG recording device in the pocket of the textile unit. This is a new and exceptional trait, but the abovementioned systems and devices require software analysis in Nubbo called Leonardo. The Nuubo company has tested multiple designs of the connecting tissue, finally choosing stretchable conductive tracks and adhesive-free usage. The latter is the biggest advantage in long-term cardiac monitoring. It is worn like a sports bra, automatically positioning electrodes tightly but comfortably against the skin in the right locations to enable high-quality data gathering. There are no sticky pads to apply or wires to adjust because of the novel silver-based textile material used for the electrodes. It is indicated for use for up to 30 consecutive days of recording ECG. Recordings can be saved in the hospital computer system for cardiologists to analyze. Different systems like Zio-Patch [43] can be used for up to 14 days of constant wear as a novel Holter monitoring but still need to be returned to the medical facility for the final data software analysis (iRhythm). The so-called “trackers” (e.g., STATS Apex Athlete) frequently used by sport professionals also require further analysis not available online [44]. The hospital-grade or ambulatory ECG transmission systems usually have leads or electrodes that are taped to different parts of the body to monitor the heart rhythm The greatest value for patients would be that the electrodes do not contain adhesives. This is the new direction for long-term cardiac monitoring.

All these ECG arrhythmia monitoring system results (FDA and CE approved) should still be consulted by a doctor or other medical professional. The diagnosis, even very precise, is far in space and time from the ongoing arrhythmic event.

### Implantable Cardiac Monitors

These FDA-approved implantable cardiac monitor (ICM) models from Medtronic (Reveal) and Abbott (Confirm) are the so-called implantable loop recorders (ILR) [45]. This technology offers cardiologists and their patients an opportunity to monitor 24/7 for potentially life-threatening arrhythmias, such as ventricular tachyarrhythmias: VT, fast VT, non-sustained VT (nsVT), VF, bradyarrhythmias and asystole. They continuously monitor the electrical activity of the heart in order to diagnose symptoms such as fainting, dizziness, and unexplained seizure-like episodes. Syncope can lead to serious injury or can be a precursor to SCD. Approximately 1.5 million people worldwide suffer from unexplained syncope. In almost 10% of patients, syncope has a cardiac cause; in 50%, a non-cardiac cause; and in 40% the cause of syncope is unknown. It is a leading cause of emergency room visits. Syncope is difficult to diagnose as syncopal episodes are often too infrequent and unpredictable for detection with conventional monitoring techniques. The ICMs are placed just under the skin of the chest area using local anesthesia during a simple outpatient procedure. Available devices weigh approximately 15 g, and they are the size of a memory stick. Later, a physician analyzes the stored information. New Medtronic and Abbott ICMs connect to external platforms and smartphones. The costs of these very useful systems are quite high: the average cost in the USA is USD 8900 (range 3000–13,000 in 2018).

Unfortunately, none of the above-mentioned wearable or implantable systems seem to have solved the problem of continuous ECG monitoring with quick reaction in case of an emergency.

## 7. Artificial Intelligence in Medicine and SCA Management

Artificial intelligence (AI) is the intelligence presented by machines in contrast to the natural intelligence of humans. The term AI is often used to describe devices that mimic cognitive functions of the human brain, such as successful learning, wise thinking, and problem solving. It has been applied to diagnosis and treatment, and it has been proven that a computer can help with clinical decision-making [45]. Some promising attempts of the AI application in OHCA have recently been made. The use of AI as a tool to improve the key components of the chain of survival is under evaluation. Recently, a machine-learning approach was used to recognize OHCA from the recordings of emergency calls to an emergency medical dispatch center, and the performance of the machine-learning framework was subsequently assessed [45]. The machine-learning-based system had a significantly higher sensitivity (84.1% vs. 72.5%, *p* < 0.001) with a slightly lower specificity (97.3% vs. 98.8%, *p* < 0.001) compared with the dispatchers. Another application of AI in terms of recognition of OHCA is the integrated software home assistant devices. Widespread use of smartphones offers a unique opportunity to identify agonal breathing and link unwitnessed cardiac arrest victims to EMS or lay people present nearby. A recent study hypothesized that existing devices (e.g., smartphones) could be used to identify OHCA-associated agonal breathing in a domestic setting. The researchers developed a specific algorithm that recognizes agonal breathing through a dataset from EMS and obtained an overall sensitivity and specificity of 97.24% and 99.51%, respectively [45]. The last example of the potential use of AI is as a tool to predict survival. Two studies reported the use of AI as a deep-learning-based prognostic system and a machine-learning algorithm to discover potential factor-influencing outcomes and predict neurological recovery and discharge from hospital [45]. Further research is needed to understand the potential of this new AI technology as a tool to support human clinical decisions.

The presented below the fuzzy logic-based heart rhythm algorithmic analysis is an example of an AI implementation in medicine due to its optimization and self-learning properties [46]. If this project is successful, its results will become a foundation for a citywide and countrywide rapid rescue system for people at risk of SCA.

## 8. Smart Implants

Smart implants (Sis) are medically implantable devices with sensors which assess the sensory input and decide on a response. An active Si is made up of biosensors, a wireless module enabling telemetry, and a miniaturized computer [46]. Examples of Sis are cardiac electronic implantable devices (pacemakers, ICDs, S-ICDs, cardiac resynchronization therapy devices) and ILRs which constantly trace the heart rhythm. Smart implants should be small, convenient for minimally invasive implantation, biomimetic, hermetic, and biostable, and their power consumption should be minimal as well. The idea of implementing the fuzzy logic-based technology for Sis is obvious. This concept resembles the one used in ILRs, but there is one crucial issue. New technology implementation should be assessed in clinical trials in terms of efficacy, but also cost-effectiveness. Therefore, the author of the PROTECTOR project concept strongly points out that the first pilot phase should be based on the I-Wear idea. Such a system is much easier to create, and work on a prototype has already commenced and the estimated budget is several times lower in comparison with Sis. The demonstrator is developed on off-the-shelf-based subunits, and the next step should be dedicated to semiconductor creation with its miniaturization for implantable versions. Implementation of the fuzzy-logic algorithm for Sis is the goal for the future.

The communication between an Si and a receiver is achieved through a medical implant communication service (MICS). The receiver then communicates via a wired network with the server, which saves long-term data. The possibilities for the communication between IMDs and/or wearable medical devices are as follows: WLAN (wireless local area network), WPAN (wireless personal area network), LR-WPAN (low-rate wireless personal area network) and WBAN (wireless body area network) [46]. The first three are more suitable for on-body communication, while the latter is better for in-body communication. Human energy harvesting or wireless powering is probably the future direction of Si battery supply. Such systems have already been developed [46].

The lifetime of batteries will always be limited and thus would require replacement through surgical interventions. Silicon remains the material of choice for micromachines applications because of its compatibility with integrated circuitry (IC) and complementary metal–oxide semiconductor (CMOS). Glass is the material of choice in IMDs that incorporate wireless interrogation.

## 9. “I-Wear”—Intelligent Wear—General Concepts

“Smart” or “intelligent” clothes—“Intelligent Wear” (I-Wear)—provide the non-invasive measurements of some basic life parameters of the human body [47,48,49,50,51]. Progress in technology enables miniaturization of sophisticated sensors and places them in wearable clothes made of new, low-cost materials. The two goals of the research are to improve the quality of the signal and to increase the number of sensors to provide more detailed information [52,53].

Most wearable medical systems are focused on non-invasive monitoring of vital signs, such as ECG, heart rate (HR), blood pressure (BP), respiratory rate, and blood oxygen saturation (SpO2). The appropriate sensors can be placed in body-worn devices, e.g., gloves, wrist-worn devices, finger rings, earlobe devices, and patches. In another, more novel approach, they are integrated into intelligent biomedical clothing. Several prototypes of the I-wear have been developed over recent years, e.g., LifeShirt, Health-Shirt, and VTAM T-shirt [54,55,56,57,58]. The HR and ECG are one of the simplest to measure and most valuable cardiovascular parameters. They can be assessed using many techniques depending on different features of the heart activity—electrical function (skin electrodes/sensors), muscle movement (microwave sensors, Doppler effect), pump function (photoplethysmography), and noise production (phonocardiography) [57]. Individual sensors of the I-Wear establish a wireless body network area (WBAN), that uses RF techniques, Bluetooth, or ZigBee to send the information to an external device or terminal for analysis and storage [58].

What still remains a big challenge is proper processing of the acquired data, especially avoiding inaccurate classifying of the rhythm-related cardiac events, which can be caused by artifacts or unsatisfactory efficient decision-making algorithms [59,60]. Different solutions have been proposed to improve the results in this field. The new concept to be used in the PROTECTOR project as a low-cost wearable device and as Si in the future is presented below.

### “I-Wear” Protector Intelligent Wear, Detailed Concept in PROTECTOR Project

**Figure 2:**
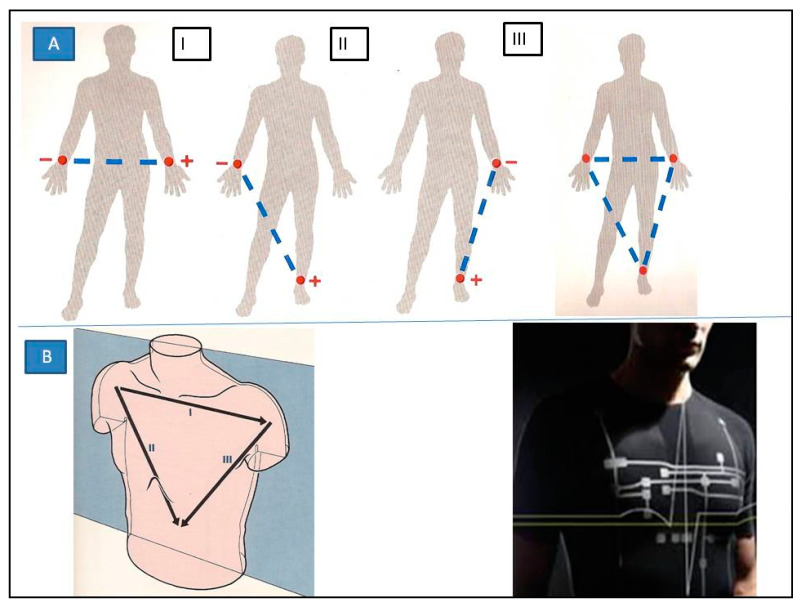
Three typical so-called “limb Leads”: I, II, III, derived from the Einthoven’s triangle (**A**) and its use in PROTECTOR Project I-Wear (**B**). The lead I is composed of the right arm, which is designated as negative, and the left arm, which is considered positive. The lead II is composed of the right arm, which is designated as negative, and the left leg, which is considered positive. The lead III is composed of the left arm, which is designated as negative, and the left leg, which is considered positive. These three standard leads form a triangle over the body as described by Einthoven( panel B).

Electrocardiogram is a process of recording the electrical activity of the heart that occurs during each contraction. A conventional ECG recording consists of 12 leads; six of them register electrical potential in the frontal plane (three typical so-called limb leads; I, II, and III, and three so-called augmented limb leads; aVR, aVL, and aVF) while the other six precordial leads “look” at the heart in the horizontal plane. Regarding the ECG signal acquisition to obtain a picture of the RR interval without evaluating morphology, it seems optimal to use traditional bipolar limb leads (I, II, and III) in the PROTECTOR project. Normally they are placed on the distal parts of the limbs near wrists and ankles, but the electrodes can be located anywhere on the limb since the potential at each point of the hands or legs is similar. The electrodes can therefore be moved to the base of the limb or to the chest near the shoulders with no significant changes to the signal quality (Figure 2). These leads form the so-called Einthoven’s triangle. By moving these leads more centrally towards the chest, Bailey created a corresponding arrangement. In the PROTECTOR garment we utilize a similar solution. We use one bipolar lead, which is a minor modification to the classic leads I, II, or III. The right lower limb in this system is used as a place for the neutral electrode, however, and it can be placed on the right side of the chest as well. The PROTECTOR I-wear ECG is obtained in the frontal plane and is presented schematically in Figure 2, panel B. We use textronic and/or metal electrodes located in the vertices of the modified Einthoven’s triangle: below the left clavicle (I), below the right clavicle (II), and in the left axillary line in the fifth intercostal space (III), and the neutral electrode is placed on the right side symmetrically to the last one. The signal (potential) is transferred to the integrated circuit embedded in the dedicated part of the I-Wear in silicon dies, called closed pocket, with a good transmission to the mobile phone. The technical and electronic details of the proposed integrated circuit are outlined below.

It is assumed that the designed I-Wear has all the advantages of typical body lingerie or elastic T-shirt: body adhesion, thermal insulation, and moisture permeability as in sportswear, comfort during normal activities and sleep, as well as physical activity including sports. It is durable and offers the possibility of standard washing. Subsequently, several improved prototype versions are developed until obtaining an optimal version, which will then be manufactured in the required quantity prior to the test phase. The garment design is different for women and men for obvious anatomical reasons, produced in typical small (S), medium (M), large (L) and extra large (XL) sizes. It is important to clearly label the shirt through the recognizable logotype of the project for medical and educational purposes, and to enhance its visibility with typical reflective elements. The place of the prototype I-Wear in the PROTECTOR system is schematically presented in the graphical abstract.

The PROTECTOR project opens an unprecedented opportunity to support any health care system through application of the unique, innovative fuzzy logic-based controller, designed to be low-cost and customizable. Thus, mobile application is planned as the front end of the PROTECTOR monitoring system. The software running on a smartphone is used to collect signals from the I-Wear garment hardware and generate alarm messages. The innovative solution consists of the local data processing and result. Wireless data transmission is limited to a radio-connection link checking periods and alert conditions when the limited amount of data is to be transferred from the garment to the mobile device. It supports the I-Wear garment power efficiency improvement, extended battery lifetime and limited radio-link load. It is an important advantage of the proposed system in comparison with already existing smartphone-based solutions with continuous real-time data transfer. Cyber-safety of all transmitted data is planned and required. The I-Wear garment electronics prototype planned for development in the frame of the project was developed using COTS (commercially available off-the-shelf) electronic components like configurable logic, analogue and mixed logic modules for signal amplification and filtering, (non)volatile memories, power supply, communication modules, microcontrollers/microprocessors etc. The hardware is selected taking into account power consumption and performance requirements to handle real-time with signal acquisition and data processing. System partitioning is the research topic to decide how to handle data processing (locally in the garment or remotely on the mobile device paired with the I-Wear garment). The PROTECTOR garment hardware is assembled on the dedicated, optionally flexible printed circuit board (PCB) substrate, optimized to fit within the limited garment volume and developed to ensure the patient/user comfort.

## 10. PROTECTOR Project. Methodology and Planned Results

The objective of the project is to develop a secure and rapid rescue system for patients at risk of SCD [61]. This system is based on the following scenario.

A patient is wearing a special I-Wear garment. If an event of cardiac arrest occurs, the electronics in the garment detects it, produces an alarm signal on the paired dedicated mobile device (audible and visible for bystanders, such as family members or people in public spaces), localizes the site of the event and transmits the alarm message (together with location) to the emergency medical center and other predefined rescue services. Prompt SCA diagnosis, audible alarm signals and alarm messages sent to the medical rescue team allow the people witnessing the event to start phone-guided CPR (medical professional-assisted) or/and AED treatment, if an AED is available nearby. At the same time, the nearest professional rescue team is dispatched to the place of the event, continues resuscitation upon arrival and, if necessary, transfers the patient to a hospital for post-resuscitation care.

This rapid rescue system requires development of the following components:

1. A wearable garment with dedicated electronic circuitry (processor) that allows the acquisition and appropriate preprocessing of the ECG signal, detection of the cardiac arrest characterized by 100% sensitivity, generation of a local audible and visible alert signal, and fast wireless transmission of the alarm message with diagnosis, the ECG signal and location information.
2. The system of reception of the alarm messages by the emergency medical staff at emergency medical centers.

### 10.1. The Fuzzy Logic and Fuzzy Rules-Based Diagnostic Algorithm in Hearth Rhythm Classification. Background

Modern science and engineering are based on mathematics and algorithmic approaches [62]. It is usually assumed that in order to solve a problem, an appropriate mathematical model must be established. Such model is a system of algebraic and/or differential equations which relate some measurable quantities to other measurable quantities. To solve a problem described by such model, a method or algorithm is needed to find a suitable solution of this system of equations. However, there are problems that cannot be solved in this way: problems for which mathematical models are not known and problems described by models which are so complex that algorithmic solutions are impractical or even impossible. Everyday experience shows that despite lack of a model or an algorithmically acceptable model, solutions to such problems can often be found.

Fuzzy logic is a mathematical theory developed in order to deal with such imprecise rules and apply these rules in a well-defined way to problems having no ordinary mathematical models or satisfying algorithmic solutions [63]. Such problems are often encountered in biology and medicine, and arrhythmia diagnosis of the underlying heart rhythm is an example of such a complex phenomenon.

Fuzzy logic (62) is based on 2 main concepts: fuzzy set and linguistic variable. These concepts can be explained taking HR (number of heart beats per minute) as an example. This is a measurable quantity, but in practice it is often stated that HR is slow, moderate, or fast rather than giving the exact value. In fuzzy logic terms, the HR is a linguistic variable that can have 3 different values (slow, moderate, fast). Usually, HR between 60 and 90 is considered moderate, lower than 60 is slow, higher than 90 is fast. In an ordinary mathematical approach, we would say that there are three sets of values of HR: a set of slow values (all values below 60), a set of moderate values (all values between 60 and 90) and a set of fast values (above 90). Thus, HR = 59.9 would be slow but HR = 60.1 would be moderate. Such sets with sharp boundaries are called crisp sets. However, real boundaries are not sharp, e.g., a HR that is moderate for one person may be too slow for another. This leads to the concept of a fuzzy set, characterized by a membership function, which can have any value between (and including) 0 and 1. This function assigns a membership value (also called grade of membership or degree of membership) to every value of HR. The membership functions of adjacent sets may overlap, and HR values may belong partly to one fuzzy set and partly to another. Every membership function establishes a quantitative link between a linguistic variable (and a fuzzy set associated with it) and an ordinary measurable quantity. If a suitable set of fuzzy rules is defined, fuzzy logic can be used for automatic control of a physical process, for classification, diagnosis, decision-making, etc. At the inputs there are ordinary variables representing physical quantities (such as HR). In the first step, called fuzzification, all membership functions are evaluated and membership values which correspond to the values of the input variables are found. In the second step (also called fuzzy inference), all AND/OR operations are performed. The results of these operations determine the shape of the membership function of the fuzzy set assigned to the output linguistic variable. In the last step, called defuzzification, the membership function of the output fuzzy set is used to find the most appropriate value of the output variable.

To apply fuzzy logic to a particular problem, one has to define the input and output variables, the fuzzy sets and the fuzzy rules. If the fuzzy sets and rules to be used are not obvious, an optimization procedure may be employed. In this way, the diagnostic algorithm for classification of cardiac arrhythmias was developed. The highest value indicates the most probable diagnosis. This result indicates probability of a complex phenomenon such as a heart rhythm classification. Probability between and including 0.8 and 1 indicates a positive result for the diagnosis of the certain kind of the heart rhythm. The diagnosis is updated after every new RR interval.

### 10.2. Fuzzy Logic Heart Rhythm Classification Algorithm (FA) Development. Methods

From a database containing RR interval series of the recorded arrhythmia events and controls, stored in the defibrillator memory and archived on PC during systematic control visits, RR data was chosen consecutively on the basis of full data availability, i.e., RR interval series with simultaneous intracardiac electrogram (IEGM) prints [64]. Then, the following heart rhythm classification of the recorded event was assigned into one of the six diagnostic categories, by the team of medical and technical experts.

The diagnostic categories of the fuzzy logic algorithm (FA 1.0):

Category 1: VF, ventricular fibrillation (indication for shock therapy)
Category 2: VT, ventricular tachycardia (indication for antitachycardia pacing or shock therapy)
Category 3: ST, sinus tachycardia
Category 4: DAI, detection of artefacts and irregularities, including extrasystoles
Category 5: ATF, atrial and supraventricular tachycardia or fibrillation
Category 6: NT, no tachycardia or sinus rhythm

**Figure 3:**
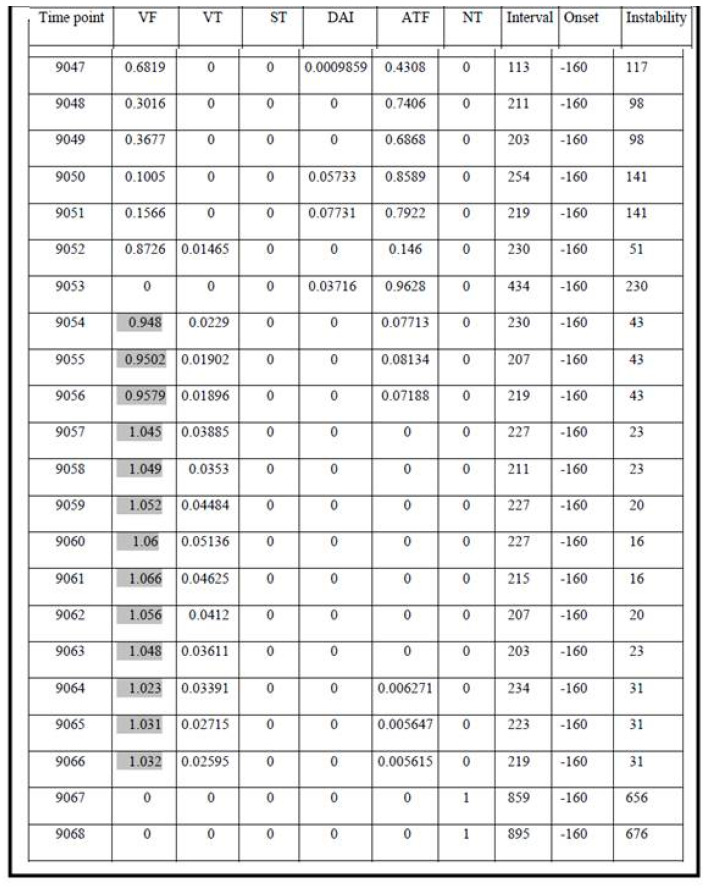
The result of FA 1.0 analysis during and following ICD discharge due to VF.

**Figure 4:**
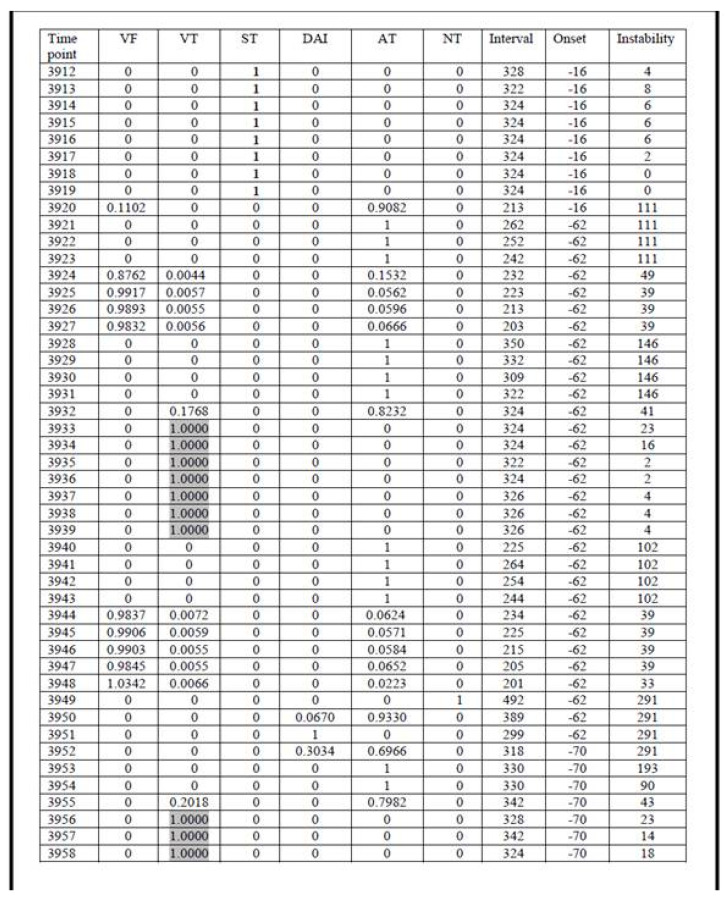
The result of FA 1.0 analysis during nsVT (non-sustained VT).

**Figure 5:**
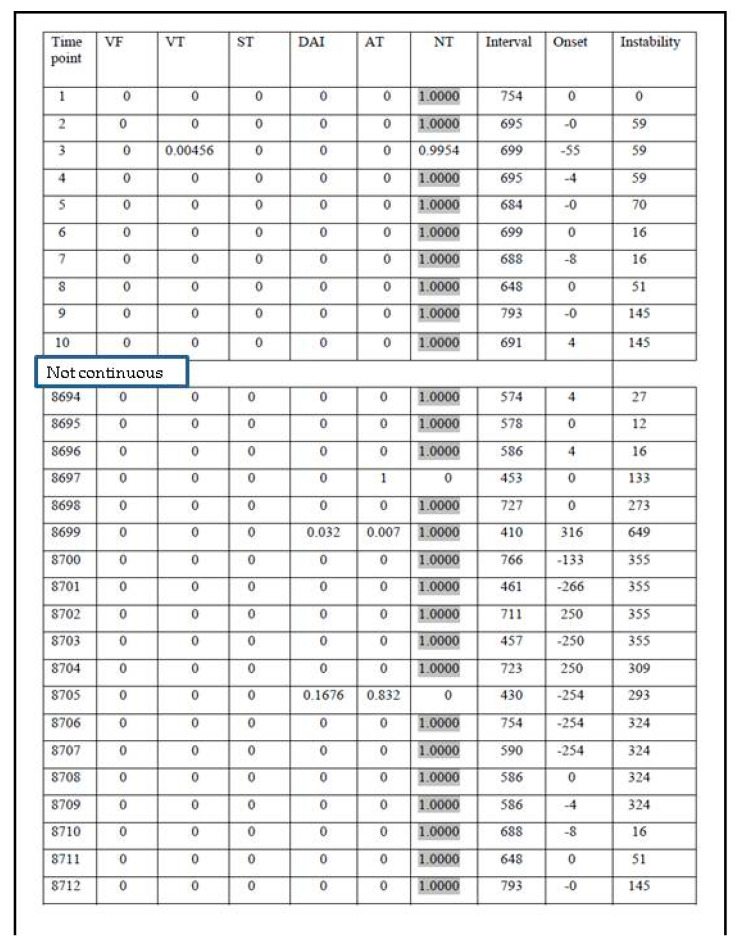
The result of FA 1.0 analysis during normal heart rhythm (sinus rhythm).

Examples of FA results in category 1, 2, and 3 are presented in Figure 3, Figure 4 and Figure 5, respectively.

The presented analysis of a certain episode consists of all possible diagnoses corresponding to each time point (column 1, Figure 3, Figure 4 and Figure 5). The columns 2–6 represent six diagnostic categories of FA 1.0. The correct FA detections are marked in the 2nd, 3rd, and 7th columns, Figure 3, Figure 4 and Figure 5, respectively.

It is well known that the analysis of the interval values alone is not sufficient to distinguish between the categories; the onset of the tachycardia (i.e., the way it begins) and its stability have also to be taken into account. For example, VT is characterized by a rapid onset and a high stability, ST by a very slow onset, DAI is extremely unstable, ATF is less stable than VT but more stable than DAI, and VF is as stable as VT and its interval values are particularly low. A HR lower than 132 bpm, which was an arbitrary lower limit of VT, suggested by two medical experts, was applied to NT category.

The RR recordings came from different single chamber ICD devices (Medtronic, Minneapolis, USA, Biotronik, Berlin, Germany, Ela Medical, Arvada, USA, Guidant, Indianapolis, USA, St. Jude Medical, Saint Paul, USA). They were further used as a basis for the definition of the optimal set of onset and stability indicators and for a fuzzy algorithm development, which is based on fuzzy rules instead of crisp threshold rules used in existing ICDs. The fuzzy logic-based approach was chosen as the methodology because it allows usage of existing qualitative knowledge and expertise in the cases when traditional mathematical algorithms fail to perform adequately or do not exist at all.

Episodes of ICD intervention and controls were analyzed and qualified by two cardiologists on the basis of the contents of the ICD Holter memory, primarily using intracardiac electrograms prior to arrhythmia detection and ICD intervention. Arrhythmia-related clinical symptoms were an additional differentiating factor. The study was approved by the National Institute of Cardiology Ethical Committee on Human Research, Warsaw, Poland.

The results were analyzed by using SPSS/PC+ statistical software (SAS v. 9.e). Quantitative variables were compared with the Student’s T test. The Fisher’s test was used when indicated (comparison of incidence of inappropriate therapies, comparison of specificity of tested algorithms). Statistical significance was set at (*p*) value *p* < 0.05.

### 10.3. Results of Fuzzy Logic Methodology for Heart Rhythm Classification in Patients with ICDs (FA 1.0)

A fuzzy logic-based control algorithm for ICDs was developed (FA 1.0) [46,64]. This algorithm proved its ability to decrease occurrence of inappropriate therapies without reducing sensitivity to life-threatening arrhythmias. A crucial proof of the effectiveness of the life-threatening arrhythmias therapy is the correct recognition of VF and VT category. The total number of RR recordings evaluated for statistical analysis came to 298 (obtained from 183 pts). Sensitivity and specificity of the proposed algorithm were calculated on the basis of the widely accepted criteria (equations are provided in the Appendix A). The sensitivity and specificity were 100% and 97.8%, respectively. Internet data transfer opens potential application of the presented heart rhythm classification methodology in telemedicine. The presented methodology is completely new ECG-based technique in the diagnosis and treatment of cardiac patients. To our knowledge, this is the first attempt of this kind of fuzzy logic implementation for this purpose. The software implementing the abovementioned algorithm was accessible for many years on a website (http://defib.imio.pw.edu.pl) (accessed on 11 October 2018). Due to formal issues, it is no longer available but during this period it was proved that internet data transfer is feasible. It is still ready to restart on a dedicated new website any time.

On the basis of the promising results described above, the PROTECTOR project team assumes that application of this methodology can be performed for SCA detection. Our studies evaluated the diagnostic value of the FA 1.0 and found it effective in recognition of supraventricular (SVT) and ventricular arrhythmias in the patients with ICD. However, in clinical practice, high sensitivity (i.e., correct VT and VF diagnosis) is accompanied by lower specificity (i.e., inappropriate treatment of SVT; rhythms like sinus tachycardia, atrial fibrillation (AF) or artefacts). Specificity of FA 1.0 seems to be sufficient for SCA detection. The correct diagnosis of life-threatening arrhythmias has to be made rapidly, within a few seconds from the beginning of the event, especially in the case of VF, as it can provoke a fatal outcome if not treated. Our algorithm meets these requirements, and it is computationally very simple. An integrated circuit which implements the FA 1.0 has already been designed.

Its computational process is repeated after every new RR interval, i.e., after intervals between sensed consecutive ventricular electrical activity—R waves. Calculation is finished in less than 100 s if the predefined number of subsequent events is smaller than the programmed value. It is possible with modern microcontrollers and 8-bit accuracy, which is sufficient for that reason. A dedicated CMOS integrated circuit implementing this algorithm has been developed as a synthesizable Very High-Speed Integrated Circuit Hardware Description Language (VHDL) model. It consists of two main blocks: the execution unit and the RAM for storage of the membership functions and fuzzy rules. In order to reduce the power consumption, clock gating is utilized, containing about 3500 sequential and about 5300 combinational gates. This circuit needs 550 clock cycles or less to produce the diagnosis with the clock frequency of 10 kHz. This is equivalent to 55 ms, well below the limit of 100 ms. The clock is switched on after every new heartbeat and switched off once the diagnosis is obtained. This demonstrator exists in the form of an internet protocol (IP) block, synthesizable VHDL code in CMOS technology. The expected average power consumption was estimated for 3 different industrial technologies: 350 nm, 90 nm, and 65 nm. The power consumption was estimated by means of Synopsys^R^ primetime tool. The dynamic power was much lower than the leakage power. The minimum total average power less than 120 MW was obtained for 90 nm low leakage process [46]. Nowadays, smartphones offer enough computing power to reliably execute this application.

### 10.4. Diagnostic Fuzzy Logic Algorithm for Sudden Cardiac Arrest (FA 2.0)

Diagnostic fuzzy logic algorithm for SCA (FA 2.0) in the PROTECTOR project recognizes only three categories:

1. Shockable rhythm—necessity to use of an AED as soon as possible (VT or VF),
2. Asystole—no electrical activity of the heart—CPR as soon as possible (no signal),
3. Normal heart rhythm (sinus rhythm)—no alert.

**Figure 6:**
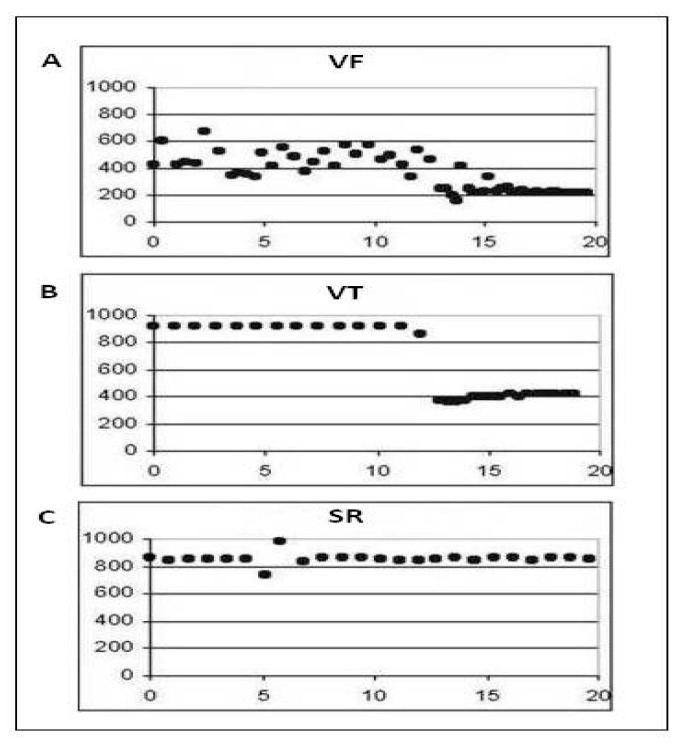
The FA 2.0 categories of the heart rhythm classification: Panel (**A**) presents the category VF, Panel (**B**) presents the category VT, Panel (**C**) presents the category normal sinus rhythm (SR). The categories VF and VT constitute new category: shockable rhythm.

The algorithm was reworked in order to limit diagnosed cardiac conditions only to those that are life-threatening. It has just 3 categories: shockable rhythm (HV), normal sinus rhythm (SR) and asystole (A). This will further lower computational complexity and power consumption for the newly developed fuzzy logic-based algorithm FA 2.0. The categories of heart rhythm classification of the FA 2.0 are presented in Figure 6.

The category “shockable rhythm” constitutes the majority of cardiac arrest cases with very high success of quickly applied defibrillation (AED). The category “asystole” encompasses a minority of cardiac arrest cases with worse prognosis, survival requiring CPR and hospital treatment where advanced life support techniques (ALS) are used. This category is diagnosed simply on the basis of no RR interval transmission and should obviously differentiate from the system OFF situation, when the user is not using an I-Wear or battery or transmission collapse occurred, etc.

The remaining approximately 10% of SCA cases, pulseless electrical activity (PEA) situations, are impossible to be detected using any ECG transmission system and they have the worst prognosis. They constitute a clinical situation with present ECG signal without hemodynamic heart pump function. The only treatment option is symptom-based CPR, BLS and ALS in the hospital [14,65]. The PROTECTOR project would enable phone-guided CPR and event location in this particular situation.

## 11. Perspectives

The diagnostic fuzzy logic-based algorithm (FA 1.0) for heart rhythm classification already exists. Its main advantages are 100% specificity and very high sensitivity, and low computational complexity leading to low power consumption. The FA 1.0 results have become the rationale for system improvements necessary to start the development of the second version of the algorithm (FA 2.0). The goal for the future of PROTECTOR project would be to design a dedicated chip (ASIC) accompanied by necessary application components to implement the diagnostic system with minimized energy consumption and fitting the minimum chassis volume. The mobile application should be dedicated to operating systems covering majority (minimum 95%) of the mobile phone/tablet market: Android (around 85%) and Apple iOS (around 13%).

**Figure 7:**
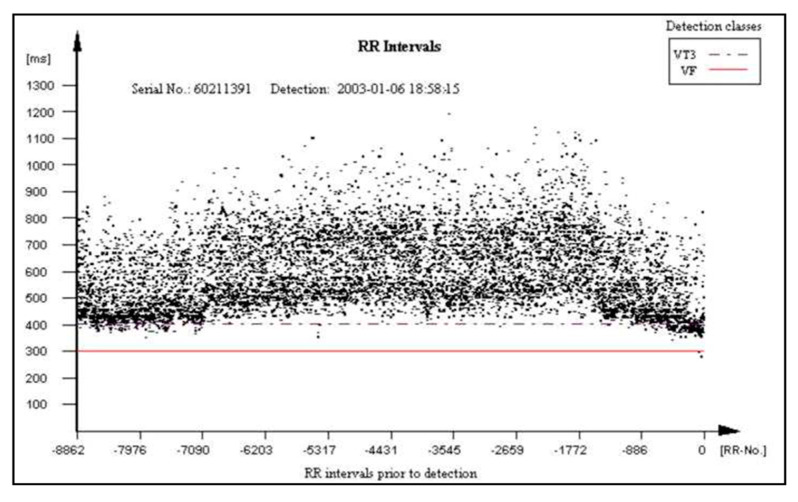
RR pattern during an episode of atrial fibrillation stored in the ICD memory.

The main aim of the PROTECTOR project is the development of the rescue system for SCA, but educational platform creation for CPR and SCA is crucial as well. The project results would be broadly implemented in the future in textile and electronic industries, telecommunication, healthcare engineering technology (Holter monitoring) and healthcare system management and optimization. The PROTECTOR application as a syncope diagnostics tool and AF detection and stroke protection (PROTECTOR-AF) tool is also possible. This diagnostic category already exists and was tested with high accuracy in ICD patients. Such arrhythmia-AF has increasing prevalence in aging societies, so its practical value of the proposed system is undeniable. The RR typical interval pattern in AF episode, which is very irregular, is presented in Figure 7.

An application ensuring athletes’ safety during sport training and mass recreational sport activities is planned as well (PROTECTOR-SPORT). For sport training and competitions, more elastic and textronic material, diminishing the influence of body movements on the ECG signal would be implemented in I-Wear. Different textiles and shapes are planned in this project.

## 12. Discussion

The global population lives longer, but with more complicated medical conditions and multiple co-morbidities (WHO data). The associated healthcare costs and increasing prevalence of chronic diseases call for new and innovative devices with advanced medical diagnostic, monitoring and therapeutic properties. Interdisciplinary research continues to push the boundaries of what is possible and develops high-tech devices. Those devices will transform healthcare as we know it today, offering a personalized and stratified approach to medicine and patients. The validation of their use in clinical trials gives these devices the opportunity to start a new era in prevention.

There are many attempts to find a good method of processing ECG signal in order to develop a diagnostic tool for classifying the arrhythmias and the heart rhythm. Some of these works have proposed fuzzy logic-based classification algorithms, often combined with another methodology like neural Network, Markov chains or wavelets, but none of these attempts seem to have solved the problem of heart rhythm accurate diagnosis [66]. The aim of the presented project is to demonstrate a new fuzzy logic-based diagnostic algorithm that combines human experience with optimization techniques. Diagnostic algorithm for implantable devices, especially for smart, small implants, must have limited computing resources and power consumption. The presented algorithm fulfills these criteria. The PROTECTOR system would instantly trace the heart rhythm, identify the person with a cardiac arrest, enable precise location of the victim, and initiate multi-pathway rescue efforts, thereby buying time necessary to preserve intact brain function. It proved to be effective in two studies using ICD data [46,64].

### Progress beyond The State-Of-Art

**Figure 8:**
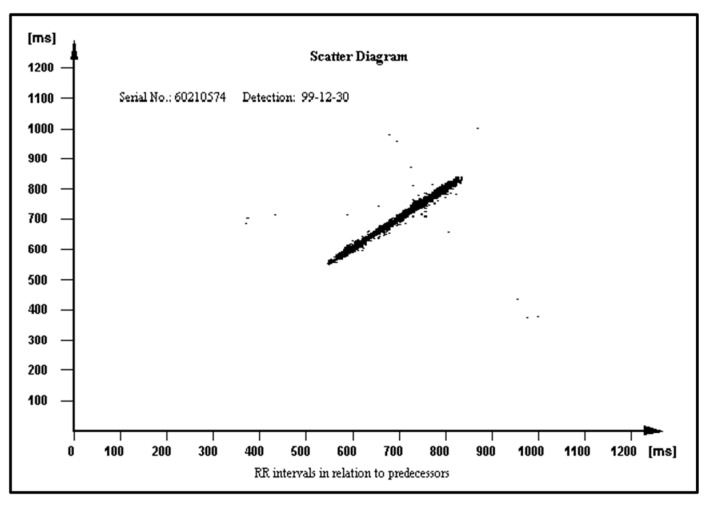
RR pattern in relation to their predecessors in normal, sinus heart rhythm.

**Figure 9:**
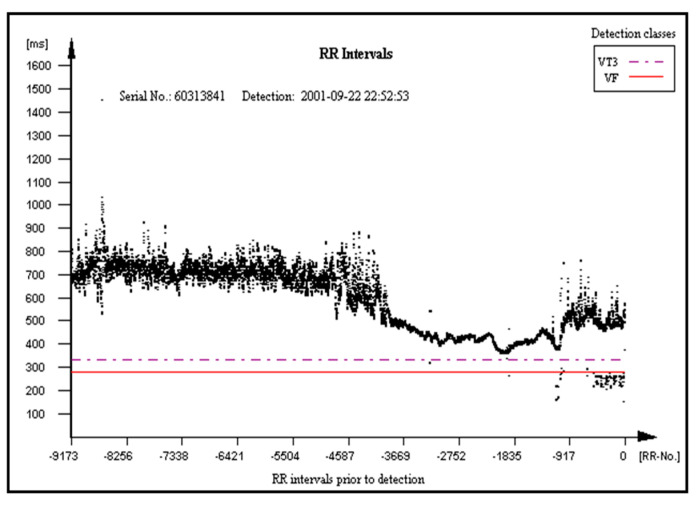
RR pattern in relation to predecessors in hypertrophic cardiomyopathy.

**Figure 10:**
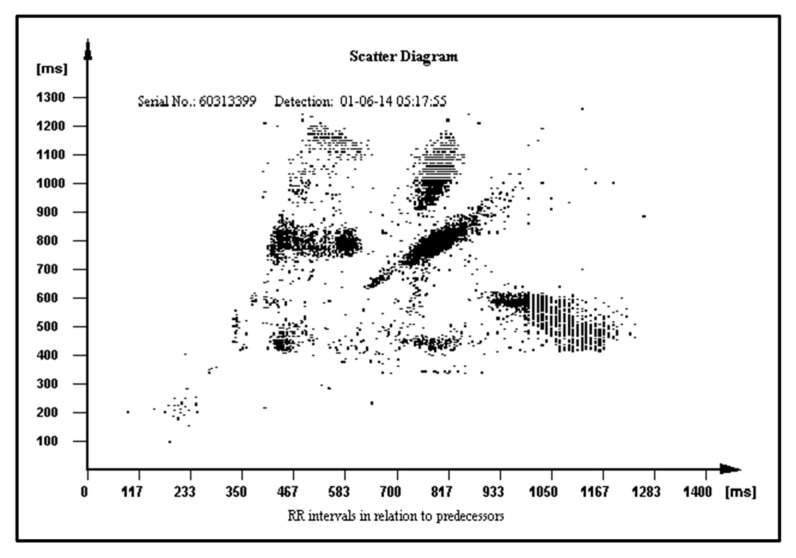
RR pattern in relation to their predecessors in ischemic cardiomyopathy.

The PROTECTOR unique feature has uninterrupted ambulatory cardiac monitoring for any period of time, and instant, precise, and automatic diagnosis on the many paired mobile devices simultaneously without the need to consult the result with the physician. The PROTECTOR does not rely on a bunch of loose wires running under the clothing to a box on the belt. Chest straps with two metal electrodes or textronic lead embedded in textile fabric seem to be better solutions than a wristband or watch due to its location on the body surface. The closer to the heart, the better the quality and amplitude of the ECG signal. This kind of I-Wear-undershirt is definitely more convenient for the patient as there is no need to remember to attach the electrodes and check the connectors. The PROTECTOR philosophy is just to have a habit to put one piece of such clothing for the day and another one for the night. This technology would become a simple and cheap method for broad sudden death risk population screening. The FA algorithm constitutes an example of a novel heart rate variability (HRV) analysis tool. The HRV was recognized in clinical trials as a SCD risk factor but is not widely used in medical practice [67,68]. Different RR patterns stored in the ICD memory in relation to their predecessors are presented in Figure 8, Figure 9 and Figure 10 in normal heart rhythm of the structurally normal hearth, hypertrophic cardiomyopathy, and ischemic cardiomyopathy, respectively.

For survivors of SCA, factors associated with poor neurological outcomes for out-of-hospital cardiac arrest include advanced age, unwitnessed arrest, no bystander CPR, time to achievement of ROSC (return of spontaneous circulation) more than 30 min, arterial pH less than 7.2, non-shockable rhythm, serum lactate levels more than 7 mmol/L, end stage renal disease and high cardiac arrest hospital prognosis (CHAP) score [11]. The methodology proposed above would influence three of the recognized factors [69,70,71,72]. The possible transfer of the RR interval recordings in a simple text format was proved in the previous study [73]. Presented algorithmic automatic diagnostic tool would be beneficial in the decision-making process in primary prophylaxis ICD implantation or even withholding the implant placement [74,75].

## 13. Summary

This paper is a comprehensive review of SCA management, wearable ECG systems, arrhythmia detection, and some technological developments in telemedicine. The newly developed fuzzy logic algorithm for heart rhythm classification and the PROTECTOR project for development of a rapid rescue system for patients at risk of SCA were widely presented. This emerging promising technology would be applicated in healthcare systems to obtain early SCA diagnosis. The presented solution would have the ability for continuous ECG tracking, which can be used in ambulatory cardiac monitoring, in sport activities and for diagnostic goals in AF and syncope. This feature enables therapy, control, and rehabilitation in cardiology but also in other medical fields. Active work in expanding knowledge is required for future developments. The educational Internet platform is an important part of the presented project. The ageing population exerts significant financial pressures on the current healthcare system. There is a need to reduce healthcare costs by minimizing in-patient hospital stays. Implementation of new monitoring technologies with communication capabilities into healthcare practice could potentially relieve the strain on the health system. It would potentially eliminate all side effects and complications of the ICD therapy by withholding implantation in some patients. The proposed wearable system and fuzzy logic implementation in Sis should decrease the rate of SCD in the future. SCD prediction is still a great challenge in medicine, and therefore there is an urgent need for effective population screening. The proposed fuzzy logic methodology for RR analysis would be such a tool. 

## Appendix A

The following equations were used for the calculation of sensitivity (i.e., appropriate ventricular arrhythmia = VT/VF diagnosis) and specificity (i.e., appropriate inhibition of potential therapy in case of appropriate diagnosis of supraventricular arrhythmia = SVT):

$$\mathrm{Sensitivity}\;=\;\frac{\mathrm{Number}\;\mathrm{of}\;\mathrm{diagnosed}\;\mathrm{ventricular}\;\mathrm{tachycardias}/\mathrm{VF}}{\mathrm{Total}\;\mathrm{number}\;\mathrm{of}\;\mathrm{ventricular}\;\mathrm{tachycardias}/\mathrm{VF}}$$

$$\mathrm{Specificity}\;=\;\frac{\mathrm{Number}\;\mathrm{of}\;\mathrm{SVTs}\;\mathrm{appropriately}\;\mathrm{not}\;\mathrm{diagnosed}\;\mathrm{as}\;\mathrm{VT}/\mathrm{VF}}{\mathrm{Total}\;\mathrm{number}\;\mathrm{of}\;\mathrm{SVTs}}$$
